# How Approaches to Animal Swarm Intelligence Can Improve the Study of Collective Intelligence in Human Teams

**DOI:** 10.3390/jintelligence8010009

**Published:** 2020-03-02

**Authors:** Lisa O’Bryan, Margaret Beier, Eduardo Salas

**Affiliations:** Department of Psychological Sciences, Rice University, Houston, TX 77005, USA; beier@rice.edu (M.B.); Eduardo.salas@rice.edu (E.S.)

**Keywords:** collective intelligence, swarm intelligence, teams, measurement, modeling

## Abstract

Researchers of team behavior have long been interested in the essential components of effective teamwork. Much existing research focuses on examining correlations between team member traits, team processes, and team outcomes, such as collective intelligence or team performance. However, these approaches are insufficient for providing insight into the dynamic, causal mechanisms through which the components of teamwork interact with one another and impact the emergence of team outcomes. Advances in the field of animal behavior have enabled a precise understanding of the behavioral mechanisms that enable groups to perform feats that surpass the capabilities of the individuals that comprise them. In this manuscript, we highlight how studies of animal swarm intelligence can inform research on collective intelligence in human teams. By improving the ability to obtain precise, time-varying measurements of team behaviors and outcomes and building upon approaches used in studies of swarm intelligence to analyze and model individual and group-level behaviors, researchers can gain insight into the mechanisms underlying the emergence of collective intelligence. Such understanding could inspire targeted interventions to improve team effectiveness and support the development of a comparative framework of group-level intelligence in animal and human groups.

## 1. Introduction

It is often said that two heads are better than one. Indeed, much of the workplace has moved towards individuals working together in teams (Ilgen 1999). Researchers within Industrial and Organizational (I/O) Psychology have long studied the properties of effective teams so that organizations can take full advantage of the cognitive and performance advantages teams can provide—a concept often encompassed by the term “collective intelligence” (Kurvers et al. 2015). Team outcomes, such as collective intelligence (and team performance more broadly), are thought to depend on a combination of team member traits and the presence and degree of team processes. For example, studies have examined how individual traits, such as intelligence, emotional intelligence, and personality, and team processes, such as communication and coordination, impact collective intelligence and team performance (Marlow et al. 2018; Marks et al. 2001; Driskell et al. 2006; Woolley et al. 2010). Team processes can be highly dynamic and can interact with team member traits, task characteristics, and team outcomes in complex and nonlinear ways over time (Kozlowski and Chao 2018). Nevertheless, in many studies, individual traits, team processes and team outcomes are measured as static representations of the team and its interactions (Kozlowski and Chao 2018). That is, these variables are often measured through methods such as self-report (i.e., retrospective surveys) and observations that capture summaries or snapshots of teamwork (Brannick et al. 1993). Thus, researchers generally have a poor mechanistic understanding (Nicholson 2012) of how individual traits and team processes produce team outcomes. In other words, while correlations between these variables have been investigated, causal explanations are largely unknown. Accordingly, developments in theory, measurement, and analysis are needed in order to clarify how individual traits and team processes promote collective intelligence, and how they are related to performance more broadly. Studies of swarm intelligence in non-human animal (hereafter “animal”) groups may provide insight due to the strong research focus on measuring and analyzing the behavioral mechanisms underlying intelligent group-level behaviors. Although animals are typically considered less intelligent than humans, they are capable of performing impressive collective feats, such as finding the most efficient route to a food source (Goss et al. 1989), choosing the highest quality nest site from a set of options (Seeley and Buhrman 1999), navigating as a cohesive group (Herbert-Read et al. 2011) and organizing a colony with division of labor (Beshers and Fewell 2000). Through studies of these impressive behaviors, evolutionary biologists have gained a detailed understanding of the mechanisms underlying successful group-level outcomes while also advancing the tools and techniques of the field. 

The goal of this manuscript is to identify areas where studies of swarm intelligence in animals can promote new insights into the study of collective intelligence in human teams. As have others (Kozlowski and Bell 2013), we define teams as groups that comprise two or more members that exist for the purpose of achieving an organizationally-relevant task. Members of teams share one or more common goals, exhibit task interdependencies and interact regularly (either face-to-face or virtually). In this paper, we start by differentiating between the concepts of collective intelligence and swarm intelligence and by adopting a definition of collective intelligence that best aligns with research in animal behavior. We move forward by providing an overview of how collective intelligence and team performance have traditionally been studied in human teams and identify limitations of these approaches. Next, we provide background on how animal swarm intelligence is studied and present two specific examples that demonstrate both the power of swarm intelligence and the associated research methods. We end with recommendations for future research approaches that can promote a more mechanistic understanding of how team member traits and interactions impact the emergence of collective intelligence in teams. 

## 2. Defining Group-Level Forms of Intelligence

Although the terms swarm intelligence and collective intelligence are sometimes used interchangeably (Krause et al. 2010), more often they are used to refer to specific forms of group-level intelligence. The term swarm intelligence tends to be used when the organisms or units under study are relatively simple, such as social insects (e.g., honey bees, ants) (Garnier et al. 2007). By contrast, the term collective intelligence is often restricted to groups of individuals with high cognitive capabilities, such as humans (Salminen 2012). However, within humans, intelligent feats generated by relatively large groups, such as online communities or crowds, tend to be referred to as “wisdom of the crowd” (Galton 1907; Becker et al. 2017) while those generated by small groups or teams tend to fall under the category “collective intelligence” (Weschsler 1971). Rather than strictly differentiating the types of groups under study, these differences in terminology (i.e., swarm intelligence, wisdom of the crowd, collective intelligence) tend to reflect differences in how these group-wide forms of intelligence are thought to emerge (Table 1).

Summary of the groups, inputs, and methods of combining inputs that commonly differentiate terms used to describe group-level intelligence.

### 2.1. Swarm Intelligence and Wisdom of the Crowd

Krause et al. (Krause et al. 2010) define swarm intelligence as situations where “group living [facilitates] solving cognitive problems that go beyond the capacity of single animals” (p. 28). Krause et al. further elaborate that swarm intelligence emerges in a self-organized manner when social interactions combine individuals’ personal information or behaviors in a manner that solves a problem better than could be done by isolated individuals (Krause et al. 2010). For example, if a school of fish is navigating towards a target and each individual moves towards an imprecise estimate of the target’s location, while also trying to remain cohesive with other group members, the group will move in the mean direction of the group members’ orientations and will thus be more likely to end up closer to the target than any individual would have on its own (Simons 2004). This example of swarm intelligence takes advantage of the ‘many wrongs principle,’ which describes group navigation contexts where the aggregate of many imprecise orientation estimates is more accurate than any given estimate (Simons 2004).

Examples of swarm intelligence that depend on the many wrongs principle are analogous to the concept of the wisdom of the crowd in human groups, which relies on the central limit theorem—a statistical property that describes how many independent samples form a normal distribution around the mean (Couzin 2009). The wisdom of the crowd has been defined as instances where “the aggregate of many people’s estimates [is] closer to the true value than all of the separate individual or even expert guesses” (Lorenz et al. 2011) (p. 1). Rather than combining individual contributions through social interactions, however, the term wisdom of the crowd refers to contexts where many independent estimates are gathered and combined by a centralized entity (Krause et al. 2010). For example, Galton (Galton 1907) famously recruited crowd members at a fair to report their best guesses for the weight of an ox and demonstrated that the median of their guesses was much closer to the actual weight than any one individual’s guess.

Although averaging all group members’ behavioral contributions is a relatively simple and common means of generating group solutions in animal groups (Sumpter 2005), natural selection has also led to the evolution of more complex ways of combining individual behaviors. For example, many feats of swarm intelligence involve individuals that display nonlinear responses to the behavior of others, such as when the likelihood that an individual will perform a particular behavior is positively and non-linearly related to the number of group members already performing that behavior, resulting in a positive feedback loop (Ward et al. 2008; Sumpter 2005; Garnier et al. 2007). Such positive feedback is often counterbalanced by negative feedback, which can aid in the stabilization of collective behaviors (Garnier et al. 2007). These processes can enable a group to become “more than the sum of its parts” (Sumpter 2005) (p. 11) and they are especially important when there are distinct differences in information possession or preferences between group members (Couzin et al. 2011; Ward et al. 2008). In sum, swarm intelligence is thought to depend on the means through which social interactions combine the behaviors of many individuals, rather than on the ability of any given individual. Thus, research on swarm intelligence centers on how group members’ behaviors and interactions promote the emergence of this intelligence, with emphasis on understanding precise causal mechanisms.

### 2.2. Collective Intelligence

Wescheler (Weschsler 1971) defines collective intelligence as intelligence which “involves, or is in some way the result of, group rather than individual mental effort” (p. 904). Wescheler further specifies that interactions between group members facilitate the emergence of insights that would not have been generated by working alone, which enable groups to produce better, or at least unique, outputs compared to individuals (Weschsler 1971). By contrast, Woolley et al. (Woolley et al. 2010) have recently reinvented the term collective intelligence by defining it in a manner analogous to individual intelligence. Specifically, they define collective intelligence as a group’s consistent performance on tasks that is correlated with (i.e., predicts) the group’s performance on other tasks. While we will discuss some studies using Woolley et al.’s (Woolley et al. 2010) definition to exemplify approaches to studying team behavior, our definition of collective intelligence better corresponds with that of Wescheler (Weschsler 1971) due to its closer alignment with the concepts of swarm intelligence and the wisdom of the crowd. That is, we define collective intelligence as the enhanced performance of groups over individuals that comes about when interactions between group members lead to the emergence of improved insights or abilities. We specifically emphasize contexts where groups enhance (rather than reduce or simply alter) individual performance to further align with definitions of swarm intelligence and the wisdom of the crowd.

Collective intelligence in small human groups or teams is thought to emerge differently than both swarm intelligence and the wisdom of the crowd for a few key reasons. Collective intelligence is considered to emerge through the cognitive abilities of individual group members when new insights are generated from the combination of one another’s knowledge, information, ideas, skills and/or perspectives (Weschsler 1971). Thus, collective intelligence is often considered to be promoted by group members with high levels of intelligence or cognitive diversity, since these traits can result in more cognitive resources (Laughlin et al. 1969; Krause et al. 2011). Indeed, Bates and Gupta (Bates and Gupta 2017) (p. 52) argue that “smart groups are simply groups of smart people.” However, since group members must coordinate and combine their individual contributions in order to take advantage of their group’s cognitive resources, the processes by which they do so can have important implications for the outcome of teamwork (Hackman and Morris 1975). Rather than interacting in a self-organized manner—where each individual is behaving without attention to a global pattern (Camazine et al. 2003)—members of human groups are typically consciously working towards a group solution and often have global (i.e., group-wide) information about the state of the group. Such access to global information can result in important differences in information transfer and decision-making processes compared to those observed in self-organized animal groups (Conradt and List 2009). For example, each group member can share their knowledge, information, ideas and perspectives with all other group members through conversation or other forms of communication. Thus, studies of collective intelligence tend to focus on how individual cognitive abilities and team processes (i.e., communication, coordination) promote the emergence of collective intelligence. However, in contrast to studies of swarm intelligence, researchers tend to have only a coarse understanding of the relationship between these variables. In the following section, we provide a brief summary of traditional approaches to the study of human teams and point to key areas that could be improved by techniques used in studies of swarm intelligence.

## 3. Traditional Approaches to Studies of Human Teams

Studies of teamwork tend to focus on how the traits of the individuals comprising a team and team processes relate to team outcomes, such as collective intelligence and team performance (Driskell et al. 2006; Woolley et al. 2010; Marlow et al. 2018). Although these studies have provided many insights into the components that are important for successfully functioning teams, research tends to focus on correlations between broad constructs. For example, the Input–Process–Output (IPO) model is a popular model of teamwork that conceptualizes how team processes (e.g., communication, coordination) mediate the effect that individual inputs (e.g., intelligence, emotional intelligence, personality) have on team outputs (e.g., performance, collective intelligence) (McGrath 1984; Hackman and Morris 1975). Accordingly, many studies have explored the relationships between IPO components (Hackman and Morris 1975).

Some studies examine the correlation between inputs, like team member intelligence levels, and team outputs. For example, studies have found that a team’s collective intelligence (conceptualized in a manner similar to individual intelligence (Woolley et al. 2010)) positively correlates with team member intelligence levels (Devine and Philips 2001; Bates and Gupta 2017). In addition, researchers have investigated inputs that are thought to affect team outputs through their impact on the effectiveness of team processes (Hackman and Morris 1975). For example, studies have reported positive correlations between collective intelligence (conceptualized in a manner similar to individual intelligence (Woolley et al. 2010)) and team members’ social sensitivities (i.e., team members’ abilities to accurately read others’ mental states) (Curşeu et al. 2015; Engel et al. 2014; Rapisarda 2002; Meslec et al. 2016; Woolley et al. 2010). Team member personality is another input that is considered to impact team outcomes through its effect on team processes (Barry and Stewart 1997; Driskell et al. 2006). For example, the proportion of highly extroverted individuals in a team has been found to negatively correlate with the level of task focus of the team (Barry and Stewart 1997), and variation in team members’ levels of extraversion positively correlates with feelings of attraction towards the group (i.e., team cohesion) (Kristof-Brown et al. 2005).

In addition to examining individual traits that serve as input to team processes, studies have investigated how team processes relate to team outputs. Team processes have been defined as “acts that convert inputs to outcomes through cognitive, verbal, and behavioral activities directed toward organizing taskwork to achieve collective goals” (Marks et al. 2001) (p. 357). Communication is one team process that has been studied extensively. Studies have found that, although communication can be positively related to team performance, the benefits of communication are contingent on both task characteristics and the type of communication involved (Hirokawa 1990; Marlow et al. 2018). For example, communication can be more important for performance on tasks that are more complex, interdependent, and that have multiple and less obvious potential solutions (Hirokawa 1990). Furthermore, communication that elaborates upon information is more important for performance than other forms of communication such as knowledge or information sharing (Marlow et al. 2018). In summary, studies of relationships between IPO components have provided insight into traits and behaviors that are important for effective teamwork. However, due to the focus on correlations between IPO components, why or how certain relationships exist is not well understood (Woolley et al. 2015).

In many studies of team behavior, input variables, such as intelligence level and social sensitivity, and process variables, such as communication, are measured in a manner that reflects their presence, type, or degree. For example, an individual’s social sensitivity has been represented by their score on the “Reading the Mind in the Eyes” test (Woolley et al. 2010) and communication is often measured based on team members’ responses to surveys of their team’s behavior (Sherf et al. 2018; Bakker et al. 2006). Furthermore, output variables, such as collective intelligence and team performance, are typically measured at the end of a team’s interaction or at discrete points throughout the interaction period. However, these approaches are insufficient for capturing the dynamics of teamwork. McGrath et al. (2000) argue that teams are complex systems, describing them as “complex entities embedded in a hierarchy of levels and characterized by multiple, bidirectional, and nonlinear causal relations” (p. 98). Even more, Kozlowski and Chao (Kozlowski and Chao 2018) describe how team processes can evolve over time based on interactions between team member traits, behaviors and changing task demands. Hackman and Morris (Hackman and Morris 1975) also describe how properties of a team and its members can both affect team processes and be affected by them. For example, team processes can lead to emergent states, such as team cohesion, which can have a positive effect on future team processes, team cohesion, and team outcomes, resulting in a positive feedback loop between these variables (Marks et al. 2001). Thus, the IPO components of teamwork can impact each other in dynamic and nonlinear ways over time. As such, simply examining correlations between input, process and outcome variables may obscure the mechanisms underlying the emergence of collective intelligence and team performance. 

By developing a causal understanding of how team member traits and team processes impact the emergence of collective intelligence and team performance, it may be possible to pinpoint how given team processes influence these outcomes over time, as well as how individual team members impact this relationship. It may also be possible to predict the collective intelligence of teams given team member traits and interaction patterns or to determine how changes to interaction patterns may impact levels of collective intelligence. In order to achieve this level of understanding, developments in research methods, theory and analysis are needed. In this regard, we can gain many insights from the field of animal behavior, which has a long history of investigating the mechanisms underlying intelligent group-level behaviors.

## 4. The Advantages of Studying Swarm Intelligence

The study of animal groups has been effective for gaining insights into swarm intelligence for a number of reasons. First, many studies focus on animals that are relatively simple, such as social insects (Krause et al. 2010; Garnier et al. 2007) or fish (Jolles et al. 2017). Since many of the group-wide phenomena under study are clearly outside the capabilities of an individual (or specifically require other individuals, as in the case of complex behavioral coordination), performance on a task must be studied by focusing on the interactions between group members. For example, army ants (*Eciton burchellii*) are capable of building bridges out of their own bodies to enable others in the colony to cross gaps in their trail. Studies of these “living bridges” examine how individual behaviors and interactions enable bridges to be constructed over gaps of various sizes, to shift location towards the most efficient path over a gap, and to break apart once traffic over the bridge dies down (Reid et al. 2015). In addition, while animals have an impressive ability to work together, their societies are comparatively simple, which promotes the extraction of the fundamental mechanisms underlying their success. 

Because we do not have access to the inner workings of animal minds, the field of animal behavior has a long history of measuring and studying behaviors (Altmann 1974). Even more, the ability to measure behavioral variables has recently been enhanced by the development of automated technologies that enable the tracking of behaviors of some or all group members simultaneously both in the lab and in the field (Kays et al. 2015; Gaëlle Fehlmann and King 2016; Graving et al. 2019b). Thus, biologists are capable of obtaining precise data on individual behaviors and interactions over extended time periods. With the aid of these data, biologists have developed both theoretical and statistical models of the dynamics underlying the emergence of swarm intelligence, which have enabled great advances in the field (Couzin and Krause 2003; Garnier et al. 2007; Couzin 2009). In addition, the processes underlying swarm intelligence in a variety of species have been found to share similar properties, and researchers have even found similarities with the cognitive processes within the human brain (Passino et al. 2008; Couzin 2007, 2009). Thus, studies of animal swarm intelligence are likely to provide insight into fundamental principles that underlie many collective phenomena, making animal groups a natural focus for insights into the collective intelligence of human teams. 

## 5. Principles of Swarm Intelligence

### 5.1. Measuring, Analyzing and Modeling Swarm Intelligence

Methodological, analytical and theoretical developments in the field of collective behavior have facilitated a precise understanding of how group-wide behaviors emerge from interactions among group members. Sumpter et al. (2012) describe how researchers can use a combination of both empirical studies and theoretical models to study individual behaviors and group-wide properties, and how these two levels of analysis connect with one another. This process is facilitated by the collection of detailed time-varying data on individual behaviors, interactions, and group properties. This level of detail is important because many social behaviors involve dynamic interactions between individuals over time. For example, many behaviors are socially contagious (Giganti and Zilli 2011; Hare et al. 2014; Reby et al. 1999). By provoking similar behavior in adjacent individuals, a display by one or a few individuals can rapidly propagate throughout an entire group. In addition, many animal decisions are known to be mediated by quorum responses, where individuals display nonlinear reactions to increasing numbers of individuals already performing a given behavior (Seeley and Visscher 2004; Walker et al. 2017; Bousquet et al. 2011; Sumpter and Pratt 2009). In order to capture these dynamics, frequent or continuous measurements of these behaviors must be obtained. Progress in this area has been aided by new technological advances in data collection that enable the acquisition of precise spatiotemporal data on the behavior of many group members. Some examples of this technology include software that can characterize individual trajectories (Moussaïd et al. 2010; Herbert-Read et al. 2011) or poses (Graving et al. 2019a) from video data and tracking devices that monitor the behavior, relative positions, and/or vocalizations of free-ranging animals (King et al. 2012; O’Bryan et al. 2019; King et al. 2018; Gaëlle Fehlmann and King 2016).

With detailed data on individual behaviors, interactions and outcomes, researchers can use data-driven approaches to analyze both individual behaviors and group-wide properties. For example, in their study of how army ants use their bodies to create bridges over gaps in the trail, Garnier et al. (Garnier et al. 2013) examined correlations between the duration in which an ant remains part of a bridge and variables such as the flow rate of ants over the bridge and the ant’s social caste. Analyses of individual behaviors such as these enable researchers to gain insight into the behavioral “rules” that underly the emergence of group-wide outcomes. In another study of bridge-building behavior, Reid et al. (Reid et al. 2015) focused on group-wide properties by measuring the length and width of the bridge over time as well as how far it shifted across an ever-widening gap. Analyses of group properties such as these enable researchers to track the progression of the group outcome over time and gain insight into important group-wide properties that may have functional consequences for the group.

In addition to collecting data on real groups, researchers also use theoretical models to explore how the aggregate of many individual behaviors and interactions result in group-wide properties. For instance, individual-based models are a popular means through which to explore how individual behaviors scale up to create collective patterns (Gueron et al. 1996; Perna et al. 2012). Once models are validated by comparing the group-wide properties of a simulated group to those of real groups, researchers can systematically alter individual behaviors or interactions within the simulation model to explore how variation in key parameters affects performance. Such models can be used to generate new predictions for how groups may respond in specific contexts, which researchers can then test by collecting new data on real groups. Should multiple potential models exist for a given phenomenon, researchers can compare their fit to real data through model selection (Farine et al. 2016; Sumpter et al. 2012). In the following sections, we provide two detailed examples of how researchers have applied these techniques to the study of collective behavior and swarm intelligence followed by a discussion of how similar approaches could be used in the study of collective intelligence in human teams. 

### 5.2. Honey Bee Nest-Site Selection—A Focus on Processes 

Studies of honey bee nest-site selection exemplify how gaining a mechanistic understanding of processes like communication can facilitate a deeper understanding of how individual communication behaviors are combined into a group-level outcome. Honey bee nest-site selection is one of the best-studied animal models for collective decision making that emphasizes the key role of communication between group members (Seeley and Buhrman 1999). Occasionally, honey bee colonies must move to a new nest site, which is a critical transition that can affect the well-being of the colony. In order to find the best nest site to transfer to, information on the qualities of a variety of potential nest sites must be collectively processed. Even though the majority of bees in the hive never visit any of the sites, and most of the bees that do visit sites only visit one (Camazine et al. 1999), groups are consistently capable of coming to consensus on the best nest site (Seeley and Buhrman 1999). Key to this process is the waggle dance—a well-studied signal that enables scouts searching for new nest sites to provide information about the location and quality of potential sites to groupmates back at the hive (Von Frisch 1967).

Important individual behaviors that facilitate the decision-making process of honey bees are the fact that the intensity of a scout’s dance positively correlates with the perceived quality of a nest site, the likelihood that other scouts are attracted to a given site positively correlates with others’ dance intensities for that site and the intensities of all dances decay at a consistent rate over time (Seeley 2003; Kameda et al. 2012). This means that a positive feedback loop exists where more scouts are recruited to high quality sites, who then return to recruit even more scouts. Furthermore, because individual dances for lower quality sites both die out more quickly and have a lower rate of recruitment than dances for high quality sites, a negative feedback loop causes support for lower quality sites to decline relatively rapidly (Seeley 2003). By video recording dancing behavior over time, researchers can track the progression of the decision-making process. Indeed, studies indicate that, although an array of site options are supported through waggle dances early on in the decision-making process, the decision is usually distilled to only two options and then finally one during later stages of the decision-making process (Makinson et al. 2014). The decision point for when to leave the current nest and move towards the chosen site is controlled by quorum sensing. That is, hive transfer is triggered when the accumulation of a sufficient number of scouts is perceived at one of the nest sites (Seeley and Visscher 2004). Thus, while much of the honey bee nest selection process is self-organized, with individuals responding to their personal information and interactions with group members, key transitions in the decision-making process are controlled by individual monitoring of group-level properties (Sumpter 2005).

By understanding how specific individual behaviors lead to group outcomes, researchers have been able to use simulation models to modify the parameters of the decision-making process in order to better understand their impact on the outcome. For example, Passino and Seeley (2006) developed a simulation model that captured the key properties of the honey bee (*Apis mellifera*) nest-site selection process. Using this model, they systematically varied the level of the quorum threshold, the decay rate of dances, the tendency for individuals to explore their surroundings versus be recruited, and the relative qualities of the nest sites in order to understand the impact these variables have on the decision-making process. They found that evolution has tuned many of these parameters to balance the trade-off between the speed and accuracy of decision making. In other words, compared to the manipulated values of these parameters, the values observed in real groups promote efficient decision making while reducing the likelihood that groups make incorrect choices. This example highlights the dynamic social processes that underlie the honey bee nest-site selection process and the value of gaining a mechanistic understanding of how individual behaviors and interactions impact group outcomes. Although static summaries of this decision-making process may reveal that communication is necessary for, or correlates with, effective nest-site selection, such summaries would not enable this level of detailed understanding regarding the inner workings of this swarm intelligence and how each component of the process influences the outcome. 

### 5.3. Collective Navigation in Fish Shoals-Effects of Individual Variation

Rather than focusing on individual traits that are measured using surveys or tests, researchers studying swarm intelligence focus on inter-individual variation that results in measurable differences in behavior and interactions. In this way, individual variation can be integrated into the overall understanding of group interaction processes to explore how it impacts the outcome. This approach is well exemplified by research on how fish perform intelligent group-wide behaviors such as coordinating their behavior within schools, collectively avoiding predators, and searching for food. The impressive synchrony of cohesive animal groups, such as bird flocks and fish schools, has long been a topic of fascination (Selous 1931). Couzin et al. (2002) significantly advanced understanding of these phenomena by developing a theoretical model of how individuals’ simple responses to their neighbors’ positions and trajectories allow them to move in a coordinated manner. They proposed that each individual follows three rules: (1) move away from close neighbors, (2) align with neighbors that are at an ideal distance and (3) move towards neighbors that are too far away. Using a simulation model, Couzin et al. were able to show that, if all group members follow these simple rules, the group displays coordination of behavior similar to behavior seen in real groups. Furthermore, they found that if they adjusted the range of interaction zones in which individuals attract, align or repel, groups take on different shapes, which may have functional consequences in different settings, such as during predation or while foraging. In order to test whether these proposed rules actually reflect how real individuals interact with one another, Herbert-Read et al. (Herbert-Read et al. 2011) used video recordings of mosquitofish (*Gambusia holbrooki*) to extract data on how each individual responds to the movements of its neighbors. They verified that individuals respond to one another according to the rules proposed by Couzin et al. (Couzin et al. 2002). They also refined this understanding by discovering that individuals primarily respond to the position and behavior of their single nearest neighbor and that the alignment response was the most important for understanding the observed patterns. 

While the studies described above demonstrate how basic rules of interaction lead to the emergence of coordinated travel in fish shoals, elaborations of this understanding have revealed how consistent between-individual differences in these rules can affect performance. For example, a study by Jolles et al. (Jolles et al. 2017) examined how individual traits influence group movement dynamics and foraging performance in stickleback (*Gasterosteus aculeatus*) shoals. They focused their study on how individual differences in the traits of “sociability” and “boldness” impact how quickly a group can find food. Their measure of sociability reflects how closely individuals stay to others while swimming and their measure of boldness correlates with individuals’ tendencies to explore their surroundings. They found that individuals lower in sociability tend to swim faster, and are thus more likely to emerge as leaders, which have higher levels of influence on the direction of group motion. Furthermore, groups composed of individuals with lower levels of sociability and higher levels of boldness displayed faster group swim speeds and greater levels of goal-orientation. Accordingly, these groups found and depleted food patches more quickly than other groups. Taken together, these combinations of theoretical and empirical studies have revealed the key behaviors that contribute to patterns of coordinated group movement in fish and also how variation in these behaviors, both by individuals and the group as a whole, affects their performance. 

## 6. Going Forward: Analyzing the Collective Intelligence of Teams from an Animal Behavior Perspective

Researchers can set the stage for improving the analysis and modeling of collective intelligence by continuing to build tools and techniques for measuring team member behaviors, interactions and team outcomes and by validating these measures against known psychological constructs. The advent and improvement of new technologies could promote the collection of precise, time-varying data on team behaviors, especially on teams “in the wild”. For example, Olguín et al. (2009) have begun developing and using new technologies, such as wearable badges that record audio, movement and proximity data, to record behaviors and interactions between individuals (Tripathi and Burleson 2012; Curhan and Pentland 2007; Anmol et al. 2004; Woolley et al. 2010). In addition to wearable badges, data can be collected during observations using hand-held devices and data-coding software (Jones et al. 2018; Tejani et al. 2010), through video recordings (Jayagopi et al. 2009), or extracted from email interactions or collaborative documents (Wise 2014). Using these tools, basic behavioral measures can be collected, such as the timing, duration and directedness of speaking turns, eye gaze, face-to-face interactions, edits to online documents and email exchanges, as well as the timing and duration of team member engagement in teamwork-related tasks. In addition to behaviors and interactions, it is also important to develop frequent measures of team outcome variables (or precursors to them) so that the progression of teamwork can be tracked over time and linked to the unfolding of team processes. For example, using speech recognition technology, words and phrases related to decision making could be tracked to examine the progression of the team’s decision-making progress over time, comparable to similar manual efforts to track such behaviors (Meyers et al. 1991). If team members are working on a physical outcome, analysis of video data could be used to measure the progression of this outcome at regular intervals, similar to how researchers measured the progress of bridge building in army ants (Reid et al. 2015). In addition, software has been developed that can track the revision history of online documents over time which could enable the monitoring of team progress on collaborative documents (Nunes et al. 2008). 

In order to make use of these data collection techniques, researchers must develop means of processing high-dimensional data. This problem is a key focus of research in machine learning where researchers are developing algorithms to process speech (Deng and Li 2013), audio (O’Bryan et al. 2019), movement (Gaelle Fehlmann et al. 2017; Cao et al. 2017; Graving et al. 2019a), and text data (Khan et al. 2010), to name a few (Valletta et al. 2017). Work is also needed to align objective measures of behavior with constructs known to be important in the psychological literature (Chaffin et al. 2017). Objective measures of individual traits could be developed by examining correlations between behavioral measures and traits such as personality, intelligence, task-specific knowledge, social sensitivity, team member role, and/or demographic variables. For example, research suggests that people with higher levels of agreeableness spend more time looking at others, and a pair’s summed level of agreeableness positively correlates with the duration of their mutual gaze (Broz et al. 2012). In addition, data on conversational turn-taking, face-to-face interactions, eye gaze, or email exchanges could be used to generate interaction networks between team members. This would enable the calculation of social network measures, such as node centrality and network density, which could be related to psychological constructs, such as individual leadership and group cohesion, respectively (Chaffin et al. 2017; Wise 2014). Thus, psychologists and engineers have the potential for productive collaborations in which they can work together to design tools for measuring team behaviors, interactions, and outcomes, to process the data streams that these tools produce and to align behavioral measures with meaningful psychological constructs (Buengeler et al. 2017). 

Many studies using new tools to obtain more objective and continuous levels of data on team behaviors and interactions are lacking in their theoretical framework. For example, Tripathi and Burleson (Tripathi and Burleson 2012) were able to predict days of high and low creativity (determined from self-rated and expert-coded creativity scores) from how much physical movement and face-to-face interaction time three teams displayed within an industrial research and development laboratory. While such measurements may provide more accurate measures of the team members’ levels of movement and interaction, compared to self-reports or observations, this level of understanding does not provide insight into the mechanisms through which movement or face-to-face interactions impact creativity. For example, it is unlikely that simply telling teams to “move more” will increase creativity. In this regard, new ways of analyzing and modeling these data are required for linking individual behaviors and interactions to the emergence of team outcomes. 

### 6.1. Analysis and Modeling of the Emergence of Collective Intelligence

New ways of modeling and analyzing collective intelligence in human teams could be inspired by approaches used in studies of swarm intelligence in animals. With regards to humans, this approach has most commonly been applied to studies of human crowd behavior, where “social forces” movement models (inspired by particle physics and animal movement studies) have been highly successful in predicting crowd dynamics (Moussaid et al. 2009; Moussaïd et al. 2009). While humans are individually complex, they often respond to one another in relatively simple and predictable ways in crowd contexts, similar to the examples of fish shoaling behavior described previously. For example, many dynamics of pedestrian behaviors, such as lane formation and responses to bottlenecks, can come about simply due to individuals’ motivations to move in a particular direction and to avoid collisions with others and with structures (Moussaid et al. 2009; Moussaïd et al. 2009). Furthermore, studies have built upon this understanding by building in human-specific nuances, such as walking in a line to facilitate conversation within small groups, enabling a more precise understanding of human-specific dynamics in more relaxed movement settings (Moussaïd et al. 2010). By similarly linking human team behaviors to comparable behaviors in animal groups, researchers could not only gain inspiration for new ways of modeling and analyzing these behaviors, but also gain insight into the behaviors that follow similar social principles to animal groups and those that involve higher-level processes (Moussaid et al. 2009). 

Communication is a key process in the emergence of collective intelligence and other team outcomes (Woolley et al. 2010; Wise 2014). While humans have a more complex communication system than the honey bee waggle dance (described above), their communication behaviors can follow predictable patterns (Pentland 2007). For example, human conversational turn-taking is a dynamic process in which each individual’s likelihood of speaking is influenced by both their own motivation to speak and the timing of others’ speaking turns (Sacks et al. 1978). Such temporal dynamics are analogous to those observed in Japanese tree frogs (*Hyla japonica*) (Aihara et al. 2008; O’Dell and Nieminen 2009) and marmoset monkeys (*Callithrix jacchus*) (Takahashi et al. 2013), which have been studied using coupled-oscillator models. Adapting such models to studies of turn-taking in human teams could facilitate understanding of how the “rules” of turn-taking impact group-level properties of communication, such as node centrality and network density, which may reflect constructs such as leadership (Mullen et al. 1989) and team cohesion (Wise 2014), respectively. By systematically adjusting parameter values among “individuals” in the model to simulate teams that vary in trait (e.g., personality) composition, these models could be used to predict the interaction dynamics and aggregate speaking patterns of diverse teams, which could be tested with data on real teams. Using these methods, we may gain new insights into the group-level trait combinations that increase the likelihood that some team members will dominate a conversation while others become overpowered. This knowledge could be used to build better teams or design interventions to target anticipated inequities in speaking time. In doing so, we could better equalize speaking behaviors and, subsequently, improve team outcomes (Woolley et al. 2010).

For teams that interact over long periods, it is possible that team processes may change over time. As mentioned previously, effective team processes have the potential to positively impact the emergence of team cohesion, which can have a positive impact on future team processes, team cohesion and team outcomes (Marks et al. 2001). Thus, it may be necessary to link emergent properties of the group back to individual behaviors. This approach is comparable to how models of honey bee behavior have linked a quorum of group members at one particular nest site to individual transitions between recruiting and preparing for hive transfer (Seeley and Visscher 2004). As Wise (Wise 2014) demonstrated, measures of interactions between team members could be used to calculate network density as an objective measure of team cohesion. By linking changes in network density to changes in individual communication behaviors over time, for instance, it could be possible to examine whether the global state of the group has an impact on behavioral changes at the individual level. 

The above examples demonstrate how a team process like communication can be studied in a dynamic way to gain more insight into how this process unfolds over time and is influenced by the traits of team members. However, researchers must also improve understanding of how individual inputs are transformed by team processes into group outcomes, which is fundamental to the study of collective intelligence (Weschsler 1971). This topic could be approached by linking the expression of team member knowledge, ideas, or opinions to the output of the group. Inspiration on how to approach this challenge could be gained from studies of movement decisions in animal groups. For example, studies have examined how group member preferences in travel direction (as indicated by their positions and initiation movements) impact the final direction chosen by the group (Strandburg-Peshkin et al. 2015; Couzin et al. 2005). Since individual preferences and social influences in a movement context can be studied by analyzing individual movement trajectories, the entirety of the decision-making process can be visualized and analyzed. In a similar manner, team member contributions and team outcomes could be represented in multi-dimensional coordinate space, where team members’ contributions and the final team outcome could be compared. For example, political scientists have used this approach to visualize and compare voting ideologies in Switzerland within ideological space (Leuthold et al. 2007). Through this approach, it could be possible to study how individual contributions change over the interaction period to result in the final team output. In addition, the effect team processes have on the progression of team outcomes in coordinate space could be examined by linking changes in the outcome’s trajectory to features of team processes, such as the timing or intensity of communication behaviors. This approach is comparable to how the occurrences of vocalizations have been linked to changes in animal movement trajectories (Gall and Manser 2017; O’Bryan et al. 2019).

While the above examples have largely focused on collective intelligence that emerges though communication behaviors, similar approaches could also be used to study more task-focused team behaviors. Studies of ant colonies have long focused on the mechanisms through which self-organized division of labor allows a colony to meet its requirements and adjust to its changing needs (Beshers and Fewell 2000). For example, differential equation models have been used to model task allocation in social insects by taking into account the different thresholds individuals have for working on available tasks, the need for a given task to be completed, and communication between individuals regarding the need to complete these tasks (Kang and Theraulaz 2016). Such models could be applied to studies of teamwork with interrelated task demands, such as surgical teams or engineering design teams. Differences in team members’ roles, task-specific skill levels, personality (e.g., conscientiousness) or motivation could be represented by different response thresholds for performing available tasks. These models could be used to generate predictions for the dynamics of task completion in teams with different team compositions, task demands, and patterns of communication. Predictions could then be compared to the dynamics observed in real teams, using data collected through either traditional observational techniques or more automated means of data collection (Jones et al. 2018).

### 6.2. Principles of Group-Level Intelligence

Sumpter (Sumpter 2005) argues that the benefit of understanding behavioral mechanisms is that a given algorithm can be compared and analyzed across systems. For example, Konstantinos and King (Katsikopoulos and King 2010) developed a theoretical model of swarm intelligence where group members must make a binary choice. They used the model to examine how the size of the group and the diversity of group members’ information impacted how much better (or worse) the group performed compared to an expert. Through these methods, they were able to define a set of conditions in which it could be beneficial to work as a group rather than rely on an expert. In a similar manner, team researchers could create a simulation model of team behavior using mechanistic knowledge of how individual traits and interactions impact collective intelligence. This simulation could then be modified in systematic ways to examine how variation in individual behaviors, interactions, and/or specifics of the task impact collective intelligence. This approach could even be extended to compare the performance of mechanisms underlying different forms of group-level intelligence. For example, Rosenberg et al. (Rosenberg et al. 2016) examined the emergence of group-level intelligence in a sports prediction context. They found that a method of combining individual predictions that simulates processes involved in swarm intelligence performed better than the simple averaging of group member predictions (i.e., comparable to traditional studies of wisdom of the crowd). By extending methods such as these to a variety of challenges faced by groups, and systematically manipulating group member contributions, behaviors, and interactions, researchers could directly compare mechanisms underlying different forms of group-level intelligence and gain a deeper understanding of fundamental social principles underlying its emergence.

## 7. Conclusions

While the topic of team performance has long been studied by researchers within I/O Psychology, many improvements can be made to enhance our understanding of the emergent properties of teams which enable them to perform better than the individuals within them. Due to the impressive collective feats that many animals are capable of producing, the study of animal swarm intelligence is a prime area for inspiration into the mechanisms underlying the emergence of collective intelligence and the research approaches that reveal them. By drawing upon studies in this field to improve the measurement, modeling and analysis of team behaviors, we have the potential to gain a more detailed understanding of collective intelligence and use this knowledge to improve our teams. In addition, by adapting mechanistic models of swarm intelligence to studies of collective intelligence, a comparative framework of group-level intelligence can be developed that can provide insight into fundamental principles of group-level intelligence as well as the inputs and social processes that can maximize it in different contexts.

## Figures and Tables

**Table 1:** Differentiating Forms of Group-Level Intelligence.

	Swarm Intelligence	Wisdom of the Crowd	Collective Intelligence
**Representative Groups**	Simple animals (e.g., social insects)	Humans (non-interacting individuals)	Human small groups and teams
**Inputs**	Personal information, behaviors	Individual estimates	Individual traits (e.g., intelligence, knowledge)
**Combining Inputs**	Interactions between individuals	Combination by centralized entity	Team processes
